# New Insights into the Roles of Monocytes/Macrophages in Cardiovascular Calcification Associated with Chronic Kidney Disease

**DOI:** 10.3390/toxins11090529

**Published:** 2019-09-12

**Authors:** Lucie Hénaut, Alexandre Candellier, Cédric Boudot, Maria Grissi, Romuald Mentaverri, Gabriel Choukroun, Michel Brazier, Saïd Kamel, Ziad A. Massy

**Affiliations:** 1 EA 7517 MP3CV, CURS, F-80054 Amiens, France; alexandre.candellier@gmail.com (A.C.); cedric.boudot@u-picardie.fr (C.B.); grissimaria@gmail.com (M.G.); romuald.mentaverri@u-picardie.fr (R.M.); choukroun.gabriel@chu-amiens.fr (G.C.); michel.brazier@u-picardie.fr (M.B.);; 2 Faculty of Medicine, University of Picardie Jules Verne, F-80000 Amiens, France; 3 Division of Nephrology, Amiens-Picardie University Hospital, F-80054 Amiens, France; 4 Department of Biochemistry, Amiens-Picardie University Hospital, F-80054 Amiens, France; 5 Faculty of Pharmacy, University of Picardie Jules Verne, F-80000 Amiens, France; 6 Division of Nephrology, Ambroise Paré University Hospital, APHP, F-92104 Boulogne-Billancourt, France; 7 Inserm U1018—Team 5, CESP, UVSQ, University Paris-Saclay, F-94807 Villejuif, France

**Keywords:** cardiovascular calcification, chronic kidney disease, macrophages, monocytes, uraemic toxins

## Abstract

Cardiovascular disease (CVD) is an important cause of death in patients with chronic kidney disease (CKD), and cardiovascular calcification (CVC) is one of the strongest predictors of CVD in this population. Cardiovascular calcification results from complex cellular interactions involving the endothelium, vascular/valvular cells (i.e., vascular smooth muscle cells, valvular interstitial cells and resident fibroblasts), and monocyte-derived macrophages. Indeed, the production of pro-inflammatory cytokines and oxidative stress by monocyte-derived macrophages is responsible for the osteogenic transformation and mineralization of vascular/valvular cells. However, monocytes/macrophages show the ability to modify their phenotype, and consequently their functions, when facing environmental modifications. This plasticity complicates efforts to understand the pathogenesis of CVC—particularly in a CKD setting, where both uraemic toxins and CKD treatment may affect monocyte/macrophage functions and thereby influence CVC. Here, we review (i) the mechanisms by which each monocyte/macrophage subset either promotes or prevents CVC, and (ii) how both uraemic toxins and CKD therapies might affect these monocyte/macrophage functions.

## 1. Introduction

Cardiovascular disease (CVD) is an important cause of death in patients with chronic kidney disease (CKD), and cardiovascular calcification (CVC) is one of the strongest predictors of CVD in this population [1,2,3,4]. Cardiovascular calcification is a degenerative process characterized by the accumulation of calcium phosphate (Ca/P) salts in the form of hydroxyapatite within the intimal and/or medial layers of the vessels, and in cardiac valves. Late-stage calcification of the intima usually develops after atherosclerosis and may be responsible for coronary ischemic events. In contrast, medial arterial calcification (also known as Monckeberg’s medial calcinosis) preferentially develops along elastic fibres; it leads to vessel stiffness, and favours left ventricular hypertrophy, diastolic dysfunction, and heart failure. In patients with CKD, diffuse medial calcification of dermal and subcutaneous arterioles (referred to as calciphylaxis) can also develop and lead to thrombosis, ischemic necrosis, chronic poor wound healing, and elevated mortality [5]. Intimal and medial calcification may develop independently of each another, although both processes occur in patients with CKD [6]. Calcification of the aortic valve is also common in end-stage renal disease (ESRD) and is associated with a worse prognosis [7]. Calcific aortic valve disease is characterized by an initial sclerosis of the aortic valve, followed by a progressive thickening and calcification of the valve leaflets [8]. This gradual fibrosis and calcification of the leaflet interferes with valve cusp opening, and thus hamper left ventricular outflow [9]. Along with the traditional Framingham Heart Study cardiovascular risk factors, the existence of non-traditional risk factors may explain the high prevalence of CVC and CVD in a CKD setting. These factors include CKD mineral and bone disorders (CKD-MBD), inflammation, oxidative stress, and the accumulation of uraemic toxins (UTs). Therefore, the prevalence of CVC increases linearly with decreasing renal function and calcification occurs significantly earlier in patients with CKD than in the general population [2]. Hence, preventing or reversing CVC may improve survival in patients with CKD. However, none of the therapies tested to date have unambiguously prevented CVC. A better understanding of the pathophysiologic mechanisms involved in the development of CVC therefore appears to be of critical importance if we are to identify effective means of prevention and treatment.

Cardiovascular calcification is a complex phenomenon, the regulation of which is not fully understood. Vascular/valvular cells (particularly vascular smooth muscle cells (VSMCs), quiescent valvular interstitial cells (VICs) and resident fibroblasts) are conventionally considered to be the culprits behind the osteogenic program leading to vessel and cardiac valve calcification. In fact, CVC primarily relies on interactions between the endothelium, vascular/valvular cells, and monocyte-derived macrophages [10,11,12]. Indeed, the production of pro-inflammatory cytokines and oxidative stress by monocyte-derived macrophages is responsible for the osteogenic transformation and mineralization of vascular/valvular cells [10,11,13,14,15,16]. Infiltrated macrophages also regulate atherosclerotic plaque development, from the formation of early fatty streaks caused by macrophage-derived foam cells to the rupture of late-stage calcified plaques due to macrophage secretion of matrix metalloproteinases (MMPs) and cysteine endoproteases [17]. Interestingly, monocytes/macrophages show the ability to modify their initial phenotype when facing environmental modifications [18]. This plasticity complicates efforts to understand the pathogenesis of CVC—particularly in a CKD setting, where both UTs and CKD therapies may affect these various functions and thereby influence CVC. Here, we review (i) the mechanisms by which each monocyte/macrophage subset either promotes or prevents CVC, and (ii) how both UTs and CKD therapies might affect these monocyte/macrophage functions.

## 2. Mechanistic Insights into the Calcification of Cardiovascular Cells

Cardiovascular calcification manifests as an ectopic deposition of crystalline mineral in the form of calcium/phosphate (Ca/P) nanocrystals. The main forms of Ca/P nanocrystals identified in the vessels consist of calcium hydroxyapatite (HA) (Ca_10_(PO_4_)_6_(OH)_2_), octacalcium phosphate (OCP) (Ca_8_(HPO_4_)_2_(PO_4_)_4_ 5H_2_O) and carbonate-substituted apatite (Ca:PO_4_^3−^) (CA) [19,20]. In this report, the term “Ca/Pi nanocrystals” was used to refer to HA and its precursors.

The extremely high prevalence of CVC in patients with CKD results from an imbalance between inducers and inhibitors of the mineralization process. Fetuin-A, pyrophosphate (PPi), matrix Gla protein (MGP), magnesium and bone morphogenetic protein-7 (BMP-7) account for the most well-known inhibitors. The most important calcification inducers include hyperphosphatemia, hypercalcemia, oxidative stress products, and UTs [21]. Hyperphosphatemia is the calcification inducer most strongly associated with CVC in CKD. Calcium/phosphate deposition is a passive phenomenon that takes place when the level of calcification inhibitors released by cardiovascular cells is low. As elegantly demonstrated by Villa-Bellosta and colleagues, the adsorption of Ca/P precipitates at the surface of cardiovascular cells prompt their phenotypic conversion into osteochondrogenic cells [22]. During this process, vascular/valvular cells downregulate the gene expression of CVC inhibitors (such as MGP and BMP7) and upregulate that of bone promotors (such as BMP2, RUNX2, and tissue non-specific alkaline phosphatase (TNAP)) [23]. These osteogenic factors act to transform amorphous calcium phosphate deposits into HA displaying a well-organized, calcified, crystalline nanostructure, which consists of an amorphous crystalline background crossed by long, fibrillary crystal planes [22]. The secretion of a procalcific matrix and the release of MMPs 2 and 9 (known to promote elastin degradation) amplify this phenomenon. The Ca/P nanocrystals formed in response to the CKD background also stimulate the secretion of pro-inflammatory cytokines by resident macrophages, thereby worsening CVC [19]. Ca/P nanocrystals can be internalised by vascular/valvular cells and undergo lysosomal degradation. This phenomenon triggers a rise in intracellular calcium concentration, which may alter cellular functions and drive apoptosis. Direct crystal–cell interaction, both due to electrostatic bonds with Ca/P nanocrystals or through receptor stimulation by these crystals, may also increase intracellular calcium and alter the cell physiology [20]. High concentrations of Ca and P also promote the release of matrix vesicles (MVs) by vascular/valvular cells and macrophages. These MVs display a similar proteomic profile compared to the matrix vesicles released by bone osteoblasts and do mineralize [24]. Indeed, the phosphatidyl serine (PS)–annexin V (Anx5)–S1000A9 membrane complex of these MVs facilitates the nucleation of Ca/P nanocrystals to the cell membrane. Apoptotic bodies derived from cardiovascular cells can also act as nucleating structures for Ca/Pi nanocrystal formation [25]. Free DNA in the damaged cardiovascular tissue may also constitute a molecular scaffold for Ca/P nanocrystal formation [26].

## 3. Monocytes/Macrophages and CVC

### 3.1. Monocytes/Macrophages in a Physiological Setting

Monocytes and monocyte-derived macrophages are at the core of the innate immune system; they perform important tasks in host defence, immune regulation, and tissue repair/regeneration [27]. Monocytes originate in the bone marrow, from where they are released into the circulation after stimulation by chemokines like monocyte chemoattractant protein-1 (MCP-1) [28,29]. In humans, three monocyte subsets have been defined as a function of the cell surface expression of the lipopolysaccharide (LPS) receptor CD14 and the FcγIII receptor CD16: the classical (CD14++/CD16−), intermediate (CD14++/CD16+), and non-classical (CD14+/CD16++) subsets [30]. Most early research did not discriminate between intermediate CD14++/CD16+ and non-classical CD14+/CD16++ monocytes and grouped them together as CD16+ monocytes [28]. The classical monocyte subset is the largest of the three (accounting for 80–85% of all monocytes), whereas the CD16+ monocytes (intermediate and non-classical subsets) account for approximately 10–20% of all monocytes [30]. In contrast to classical CD14++/CD16- monocytes, CD16+ monocyte counts are low under healthy (physiological) conditions and elevated under inflammatory (pathological) conditions [28,31] such as ESRD [32]. The two CD16+ monocyte subsets are better able to produce inflammatory cytokines (such as TNF-α or IL-1β) than classical monocytes [28,33]. Therefore, the CD16+ subtypes have long been presented as “pro-inflammatory monocytes” linked to atherosclerotic disease [34,35], severe aortic stenosis [36,37] and (in non-dialyzed patients with CKD) vascular stiffness and cardiovascular events [38,39]. The new tripartite view of monocyte subpopulations emphasizes a more important role for intermediate monocytes (rather than non-classical monocytes) in inflammation [28].

Upon tissue damage or infection, monocytes are rapidly recruited to the affected site, where they can differentiate into macrophages. These macrophages can switch toward a classically activated phenotype (M1) in response to Th1 cytokines (e.g., IFN-γ) or toward an alternatively activated phenotype (M2) when exposed to Th2 factors (e.g., IL-4 and IL-13). These phenotypes represent the two ends of a broad spectrum of macrophage subsets [40]. Macrophage polarization is a highly controlled process involving specific signalling pathways and transcriptional and posttranscriptional regulatory networks. Indeed, predominantly NF-κB- and STAT1-driven activation polarizes macrophages toward the M1 phenotype, resulting in cytotoxic and tissue-damaging pro-inflammatory functions after the release of cytokines such as IL-1, IL-6, and TNF-α. In contrast, predominantly STAT3 and STAT6 activation by IL-4, IL-13 and IL-10 polarizes macrophages towards the M2 phenotype, which is associated with wound healing (M2a) and anti-inflammatory (M2b/c) activities [41].

### 3.2. Roles of Monocytes/Macrophages in CVC

#### 3.2.1. The Procalcific Actions of Monocytes/Macrophages

The pro-inflammatory cytokines released by M1 macrophages constitute an essential host defence component but can also cause important damage to the host itself. Indeed, high serum levels of inflammatory mediators are known to be associated with the greater prevalence, severity and progression of CVC in haemodialysis patients [42]. Low-grade systemic inflammation is regularly seen in patients with CKD and constitutes an independent risk factor for cardiovascular morbidity and mortality [43]. The chronic micro-inflammatory state observed in these patients is characterized by high circulating levels of TNF-α and IL-6 [44], which are associated with both CVC and cardiovascular mortality [45,46]. These data suggest the existence of a causal link between macrophage-mediated inflammation and CVC in a CKD setting. Indeed, the results of early experimental studies showed the importance of monocyte/macrophage-derived inflammatory mediators (particularly IL-6, TNF-α, oncostatin M, and IL-1β) in promoting the calcification of vascular/valvular cells [11,16,47,48]. These pro-inflammatory cytokines were shown to favour the CKD-associated osteochondrogenic transition of vascular/valvular cells and the release of MVs and apoptotic bodies able to act as Ca/P nanocrystal nucleation points [48]. The NLRP3 inflammasome pathway plays a key role in this context. This multiprotein oligomer is responsible for the caspase 1-dependent release of IL-1β, a pro-inflammatory cytokine recognized as a key biomarker and mediator of inflammatory calcification [49]. Indeed, IL1-β favours VSMC osteogenic differentiation and subsequent calcification in vitro [50,51] and aortic calcifications can be inhibited by IL-1β monoclonal antibody in LDLR-deficient mice [52]. NLRP3 inflammasome markers and caspase 1 activity are upregulated within calcified vascular lesions compared with non-calcified adjacent tissues [51] and the NLRP3 inflammasome complex is upregulated in calcifying VSMCs, resulting in increased IL-1β secretion. In vitro, the inhibition of inflammasome activation by NLRP3 RNA interference reduced IL-1β secretion and inhibited VSMC calcification.

In vitro, M0 macrophages incubated with Ca/P nanocrystals polarize into M1 macrophages that produce inducible nitric oxide synthase (iNOS) [48] and pro-inflammatory cytokines such as TNF-α [19,20], which further amplify the calcification process. A recent in vitro study reported that Ca/P nanocrystals can also activate the NLRP3 inflammasome and consequently promote the secretion of IL1-β in THP1 and murine bone-marrow-derived macrophages (BMDMs) [20]. Again in vitro, human monocyte-derived macrophages can internalize Ca/P microcrystals in vacuoles [53]. In response, the macrophages release pro-inflammatory cytokines such as TNF-α, IL-1β and IL-8, which can activate cultured endothelial cells and promote the adhesion of flowing leukocytes under shear flow. In vitro, the ability of Ca/P nanocrystals to activate macrophages is inversely correlated with their size [53]; crystals with a diameter of 1–2 µm and a pore size of 10–50 Å induce TNF-α secretion most strongly [54]. This observation suggests that tiny, isolated, Ca/P nanocrystals formed during the early stages of the pathology may be more pro-inflammatory than larger deposits resembling bone. Interestingly, the macrophage-derived production of neopterin (a biomarker of M1 macrophage activity [55]) is inversely associated with the amount of calcification in human late-stage atherosclerotic plaques [56]. This further suggests that atherosclerotic tissue with large Ca/P volumes contributes less to the inflammatory burden than tissue with limited Ca/P volumes. Lastly, it should be noted that the small punctate calcifications observed in early-stage atherosclerosis also promote the secretion by VSMCs of macrophage chemo-attractants, thus feeding the inflammation-driven osteochondrogenic VSMCs transition [57]. These macrophages’ responses to Ca/P nanocrystals suggest that the pathological process of calcification is not solely a passive consequence of chronic inflammation but may trigger a positive feedback loop by which inflammation promotes CVC and CVC then promotes inflammation and drives disease progression.

In CKD, the accumulation of pro-inflammatory cytokines such as IL-6 decreases the synthesis of fetuin A by the liver. Fetuin-A is a circulating protein that binds insoluble mineral molecules to create soluble colloidal calciprotein particles (CPPs). Fetuin-A-containing CPPs prevents Ca × P product precipitation and promotes the clearance of mineral nanocrystals by phagocytic cells, thereby avoiding their deposition in ectopic locations [19,58,59,60]. Interestingly, vascular/valvular cells can also take up fetuin-A from the circulation and load it into MVs, where they can bind minerals to prevent further growth [61]. In vitro, CPPs triggered lower cytokine secretion by macrophages than HA crystals of equivalent size and calcium content [19]. Furthermore, CPPs only had a moderate impact on macrophage viability and apoptosis—even at high levels—when compared with HA crystals, which showed a strong pro-apoptotic effect at much lower levels. In a CKD setting, one should not neglect the role played by the insoluble CPPs in the vicious circle of inflammation and calcification [59].

**Figure 1:**
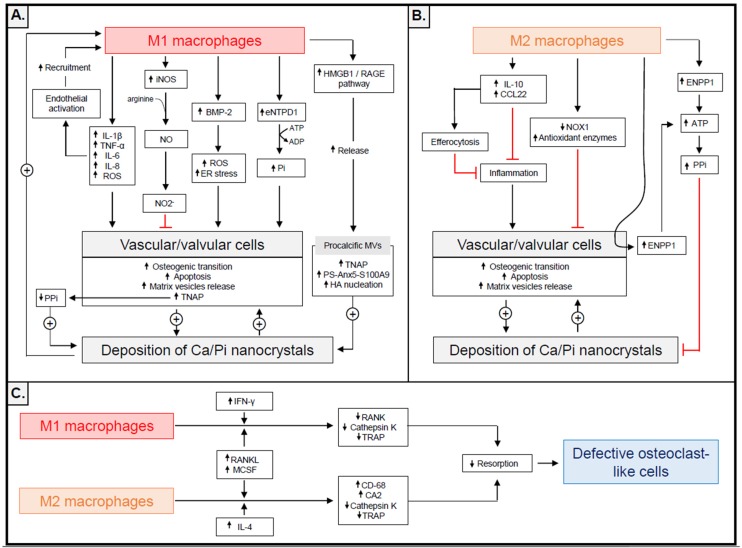
Summary of the mechanisms by which macrophages subtype may influence mineral deposition. (**A**). Mechanisms by which M1 macrophages may promote CVC. (**B**). Mechanisms by which M2 macrophages may protect against CVC. (**C**). Impact of macrophage polarization on the formation of osteoclast-like cells. Anx 5: annexin 5, BMP-2: bone morphogenetic protein 2, CA2: carbonic anhydrase, CCL22: C-C motif ligand 22, ENPP1: ectoenzyme nucleotide pyrophosphatase/phosphodiesterase-1, eNTPD1: ectonucleoside triphosphate diphosphohydrolase 1, ER: endoplasmic reticulum, HA: hydroxyapatite nucleation, HMGB1: high–mobility group box 1, IFN-γ: interferon γ, IL-1β: interleukin-1β, IL-6: interleukin-6, IL-10: interleukin-10, iNOS: inducible nitric oxide synthase, MCSF: macrophage colony-stimulating factor, MVs: matrix vesicles, NO: nitric oxide, NOX: NADPH oxidase, Pi: inorganic phosphate, PPi: pyrophosphate, PS: phosphatidyl serine, RAGE: receptor of advanced glycation end-products, RANKL: receptor activator of nuclear factor kappa-B ligand, ROS: reactive oxygen species, TNAP: tissue-nonspecific alkaline phosphatase, TRAP: tartrate-resistant acid phosphatase, TNF-α: tumour necrosis factor α.

In human carotid plaques, macrophages accumulate at sites of calcified vesicular regions. In 2013, New and colleagues hypothesized that macrophages contribute directly to microcalcification through the production of MVs [14]. The researchers reported that the macrophages released MVs of 30–300 nm when cultured in vitro. After Ca/P stimulation, these MVs displayed increased alkaline phosphatase activity and HA nucleation. Macrophage MVs contain exosomal markers (CD9 and TSG101), S100A9, PS and Anx5. In this respect, the macrophage-derived MVs were similar to chondrocyte-derived MVs that require PS and Anx5 for growth plate mineral nucleation [62,63]. According to New and colleagues, Ca/P stimulation of macrophages in vitro strengthened the association between S100A9 and Anx5 and Ca/P-stimulated macrophages externalized PS, suggesting that Ca/Pi nucleation may take place on the MV outer membrane. Silencing S100A9 expression in vitro and S100A9^−/−^ gene knockout in the mouse are associated with low MV mineralization, whereas stimulation with S100A9 increases the calcification potential. Taken as a whole, these data indicate that PS–Anx5–S100A9 membrane complex helps to promote HA nucleation within the membrane of the macrophage-derived MV [14]. In New and colleagues’ study, the Ca/P-stimulated macrophages expressed relatively high mRNA levels of the M1 markers iNOS and IL-6, while M2 markers MRC1 and ARG1 tended to decrease, suggesting that these procalcific MVs are primarily released by M1 macrophages. In a later study, high mobility group box 1 (HMGB1), a nuclear protein secreted by stressed cells [64,65] and known to accumulate in areas of macrophage infiltration and calcification in calcific aortic valve stenosis [66], has been identified as a potential inducer of MV secretion by macrophages [67]. In this study, HMGB1 signalled through receptor of advanced glycation end-products (RAGE) to promote p38 mitogen-activated protein kinase (MAPK)/nSMase2-mediated secretion of MVs from murine macrophage-like cells RAW264.7, which subsequently participated in mineralization both in vitro and in vivo. The mechanisms by which M1 macrophages may promote CVC are summarized in Figure 1A.

Osteoblast precursors are usually considered to reside exclusively in the bone marrow mesenchymal stem cell compartment. However, increasing evidence revealed the existence osteoprogenitors within the systemic circulation [68,69]. In particular, Fadini and colleagues demonstrated in 2011 that a small proportion of circulating monocytes (1% of the total, in healthy subjects) expresses bone-specific alkaline phosphatase (BAP) and osteocalcin (OC) [70]. Given that these OC^+^BAP^+^ cells (i) show the upregulation of Col1a1, Osterix, and Runx2 and (ii) calcify when cultured in vitro, and (iii) favour ectopic calcification when implanted in nude mice, Fadini and colleagues referred to this subset as “myeloid calcifying cells” (MCCs), and hypothesized that CVC results from high blood counts of these cells. Confirming their myeloid origin, these cells do not express the mesenchymal stem cell markers CD90, CD44, and CD29 but rather express CD45, CD14, and CD68, as well as BCR-ABL when sorted from naïve patients with chronic myeloid leukaemia. Osteocalcin and BAP expression in MCCs appears to be driven by Runx2, and signalling pathways involved in inflammation, bone mineral metabolism and osteogenesis are upregulated in those cells compared to OC^-^BAP^-^ [70,71]. Interestingly, MCCs isolated from mice spleen show transendothelial migration capacity and procalcific activity in vitro. Injection of those cells in ApoE-/- mice promotes calcification of early and advanced atherosclerotic lesions via paracrine activity and overexpression of the macrophage activation marker allograft inflammatory factor (AIF)-1 [72]. Myeloid calcifying cells are overrepresented in the blood of patients with type II diabetes and in atherosclerotic lesions [70]. Glycaemic control was associated with the normalization of the MCCs count in patients with type II diabetes, whereas exposure to elevated glucose concentration enhanced MCCs calcification in vitro. Taken as a whole, these data suggest that diabetes increases the bone marrow’s generation and release of this monocyte subtype, which then would home to vascular disease sites and promote ectopic calcification. To date, the putative presence of MCCs in patients with CKD has not been investigated.

More recently, Dube and colleagues reported that M1 subtype macrophages differentiated in vitro from mice BMDMs display their own osteogenic properties through constitutive activation of BMP-2-dependent signalling [73]. This phenomenon may have major pathological consequences, since BMP2 accumulation within the arterial wall promotes the procalcific differentiation of vascular/valvular cells by inducing oxidative stress and runx2 expression [74]. In line with this hypothesis, an early study reported that the exposure of human mesenchymal stem cells to conditioned media prepared from murine J774.A.1 macrophages increased TNAP expression, and that the latter effect was blocked by anti-BMP2 antibodies [75]. Future studies will be needed to confirm the involvement of macrophage-derived BMP2 in the osteogenic differentiation and calcification of vascular/valvular cells.

#### 3.2.2. Anticalcific Actions of Monocytes/Macrophages

In contrast to M1 macrophages, M2 macrophages seem to protect against CVC (Figure 1B). Indeed, co-culture of mouse VSMCs with M2 macrophages in Transwell^®^ systems significantly reduced calcium phosphate deposition, whereas co-culture with M1 macrophages had no effect [76]. There are several explanations for this protective effect. Firstly (and as discussed above), M2 macrophages do not secrete high levels of pro-inflammatory mediators; this may limit the cells’ procalcific properties when compared with M1-polarized cells [77]. Secondly, and as elegantly described by Villa-Bellosta and colleagues, M2-polarized macrophages are better able to synthesize calcification inhibitors such as PPi in vitro [76]. PP_i_ is a direct inhibitor of calcium phosphate deposition; it is produced after adenosine triphosphate (ATP) hydrolysis by ectoenzyme nucleotide pyrophosphatase/phosphodiesterase-1 (eNPP1) and is then degraded to P_i_ by TNAP. In Villa-Bellosta et al.’s study, alternatively activated M2 macrophages increased extracellular PP_i_ levels in vitro through increased ATP release and eNPP1 overexpression. In a Transwell^®^ co-culture system, the presence of M2 macrophages induced eNPP1 expression in VSMCs, and thus contributed to the accumulation of PP_i_. In contrast, Villa-Bellosta and colleagues observed the strong expression of ectonucleoside triphosphate diphosphohydrolase 1 (eNTPD1, known to produce Pi from ATP hydrolysis) by M1 macrophages; this may have been responsible for the reduction of extracellular PP_i_ due to a decreased ATP level. In this study, the M1 macrophages also induced TNAP expression in VSMCs, which would also have reduced the extracellular accumulation of PP_i_ [76].

As mentioned above, the apoptotic bodies and MVs released by calcifying cells can serve as a scaffold for the nucleation of Ca/P nanocrystals. Within atherosclerotic plaques, apoptotic cells are normally cleared by M2 macrophages (through the release of IL-10 and TGF-β). This process of ingestion of the apoptotic cells by macrophages, known as efferocytosis, allows the removal of apoptotic cells before disruption of membrane integrity and release of pro-inflammatory contents. Although this mechanism prevents the accumulation of apoptotic debris in early-stage atherosclerosis, efferocytosis is impaired in late-stage lesions. This impairment contributes to the accumulation of apoptotic debris, further activation of the inflammatory cascade, progression of the atherosclerotic lesion, and growth of the necrotic core [78]. Interestingly, early atherosclerotic lesions in a model of defective macrophage efferocytosis showed accumulation of calcium deposits [79]. These findings prompt the hypothesis whereby (i) the M2-mediated engulfment of apoptotic residues may prevent Ca/Pi nucleation in early-stage atherosclerosis, and (ii) this mechanism is weakened by a gradual decrease in the effectiveness of efferocytosis. Further studies will be needed to definitively establish whether or not there is a causal link between defective macrophage efferocytosis and the development of CVC.

#### 3.2.3. Monocytes/Macrophages as Precursors of Osteoclast-Like Cells

Calcified cardiovascular lesions contain osteoclast precursors (i.e., monocytes/macrophages) and osteoblast-like VSMCs capable of secreting factors involved in osteoclast differentiation (such as receptor activator of nuclear factor kappa-B ligand (RANKL) and macrophage colony-stimulating factor (MCSF)) [10,80,81]. In vitro, RANKL expression by VSMCs induced the osteoclastic differentiation of monocytic preosteoclasts (RAW264.7 monocytes-macrophages or mice BMDMs in co-cultures)—an effect that was blocked by the addition of osteoprotegerin, a soluble decoy receptor for RANKL [10,82]. This suggests that a process resembling osteoclastogenesis occurs in the cardiovascular wall. In line with this observation, tartrate-resistant acid phosphatase (TRAP)-positive multinucleated giant cells, with a morphology closely resembling that of osteoclasts, have been observed at site of Ca/P deposition in human atherosclerotic lesions [83,84]. In atherosclerotic plaques of human carotid arteries, these cells were positive for CD-68 (a typical surface marker for macrophages) and displayed high expression of the osteoclast-associated antigens cathepsin K, receptor activator of nuclear factor kappa-B (RANK), and osteoprotegerin [85]. Initially, the presence of TRAP and cathepsin K (both enzymes associated with bone resorption) suggested that these osteoclast-like cells might be able to resorb calcified vascular lesions. This hypothesis was recently reinforced by the observation that mature osteoclasts actively reduce the mineral load of pre-calcified aortic elastin in vitro [86]. Furthermore, macrophages reportedly express high levels of carbonic anhydrase 2 (CA2) [87], an enzyme used by osteoclasts to dissolve minerals by creating an acidic environment through H^+^ production in the resorption lacuna [88,89,90]. In an in vitro cell-free assay of ectopic calcification, the level of CA2 expression was correlated with the macrophages cell lines’ decalcification activity [87]. Cathepsin K is expressed in late-stage lesions but not in early-stage lesions. In vitro, human monocyte-macrophage foam cells generate CTX-I fragments when cultured on collagen type I-rich matrix, and this effect is blocked by the cathepsin K inhibitor E64. By degrading collagen type 1 (a key substrate for the formation/fixation of calcified nodules), macrophage foam cells may therefore prevent the initial development of calcification within the early atherosclerotic plaque [91]. However, the osteoclast-like cell count within calcified vascular lesions is low, and CVC rarely regresses in vivo. This suggests that certain factors in the vessel wall decrease the differentiation and resorption potential of osteoclast-like cells. In line with this hypothesis, calcifying vascular cells were shown to block osteoclast differentiation in vitro through increased Il-18 secretion [92]. Similarly, Mazière and colleagues reported that in vitro exposure to oxidized low-density lipoprotein (oxLDL) prevented RANKL-induced TRAP activity and the subsequent bone resorbing activity of human peripheral blood mononuclear cells (PBMCs) [93]. In the latter study, oxLDL also prevented the RANKL-induced generation of multinucleated osteoclast-like cells from mouse RAW 264.7 monocytes-macrophages. Furthermore, N-acetylglucosamine-1-phosphate transferase, alpha and beta subunits (GNPTAB), a trans-membrane complex known to catalyse the synthesis of mannose 6-phosphate recognition markers on certain oligosaccharides of lysosomal enzymes, recently emerged as a potential modulator of the osteoclast-like cells’ hydrolase activity. Osteoclasts derived from the bone marrow of GNPTAB-deficient mice have elevated levels of TRAP and cathepsin K [94]. In vitro, GNPTAB silencing with a siRNA increased lysosomal hydrolase expression and improved the function of osteoclasts derived from human PBMCs [95]. The subsequent observation of higher GNPTAB expression and lower cathepsin K and TRAP expression in human calcified atherosclerotic plaques (relative to non-calcified areas) [95] confirmed that GNPTAB may be one of the factors responsible for the dysfunction of vascular/valvular osteoclasts.

Recent data have suggested that macrophage polarization also contributes to low osteoclastic activity in human calcified atherosclerotic plaques [96]. In a recent study, Chinetti-Gbaguidi et al. showed that macrophages surrounding the Ca/P deposits in human atherosclerotic plaques express CA2 and a relatively low level of cathepsin K [97]. These macrophages also expressed the mannose receptor (CD206)—a marker typically expressed by activated M2 macrophages. The subsequent observation that monocytes differentiated in vitro toward the M2 phenotype via exposure to IL-4, RANKL and MCSF displayed low levels of cathepsin K expression, low TRAP activity, and low bone matrix degradation activity, suggests that a polarization toward the M2 phenotype renders macrophages ineffective to resorb the calcification. Among the molecular mechanisms involved, the authors showed that IL-4-induced polarization lowers the expression level of the cathepsin K transcriptional regulator nuclear factor of activated T cells type c-1 (NFATC1) as well as its induction by RANKL/MCSF, through upregulation of NFATC1 promoter level of the repressive histone 3 lysine 27 trimethylation (H3K27me3). According to the authors, IL-4 induced the inhibition of the ERK-c-fos-NFATc-1 pathway and may also be responsible for the impaired mineral resorption of these osteoclast-like cells [96]. In the same manner, Nagy and colleagues studied the impact of M1 polarization on the resorbing capacity of cardiac valve monocytes. In this study, the in vitro treatment of peripheral blood CD14+ monocytes with IFN-ɤ led to morphologically and functionally defective osteoclasts [98]. Data obtained ex vivo demonstrated that the release of IFN-ɤ from activated cytotoxic T cells blocked the formation of osteoclast-like cells within cardiac valve tissue and was followed by an increase in valvular calcium content. In line with this observation, a subsequent study by Barinda and colleagues evidenced the negative regulation of CA2 expression in RAW cells polarized toward the M1 phenotype [87]. The researchers found that treatment of RAW cells with vasoactive peptides (such as endothelin-1 and angiotensin II) or inflammatory cytokines (such as TNF-α and IL-1β) also reduced CA2 expression [87]. Taken as a whole, these results indicate that exposure to either a pro-inflammatory or anti-inflammatory environment makes the macrophages around vascular Ca/P deposits phenotypically defective and thus unable to resorb calcification (Figure 1C). In this context, new pharmacological techniques for enhancing the osteoclastic activity of macrophages might prompt the development of exciting cell-mediated therapies that can prevent or resorb CVC.

## 4. The Impact of CKD on Macrophage Functions: Consequences for CVC

### 4.1. Uraemic Toxicity and Monocyte/Macrophage Functions

#### 4.1.1. Influence of CKD on Monocyte Subtypes

A compelling set of evidence suggests that the uraemic environment in CKD predisposes to vascular inflammation and subsequent calcification via (i) elevated monocyte adhesion, rolling and extravasation, and (ii) direct modulation of the monocytes’ and macrophages’ pro-inflammatory potential. Indeed, the proportion of pro-inflammatory CD14+/CD16+ monocytes is abnormally high in both dialysed and non-dialysed patients with CKD [99]. In pre-dialysis patients, this elevated CD14+/CD16+ cell count is closely associated with levels of high-sensitivity C-reactive protein and IL-6 (markers of the systemic inflammation observed in CKD) [38]. Compared with CD14++/CD16- cells, CD14+/CD16+ monocytes express a pro-atherogenic pattern of chemokines and adhesion molecules, such as CX3CR1 and ICAM-1 in patients with CKD [100]. CD14+/CD16+ cells collected from patients with CKD adhered more strongly to a human umbilical vein endothelial cell monolayer in vitro than the corresponding CD14++/CD16- cells did [100]. The elevated CD14+/CD16+ monocyte count in patients with CKD was positively correlated with the presence of apoptotic endothelial microparticles, suggesting that a link exists between elevated CD14+/CD16+ and endothelial dysfunction in CKD [99]. Experimentally, CD14++/CD16+ monocytes isolated from donors with CKD displayed preferential lipid accumulation, high expression levels of CD36 and CD68, and low expression levels of the cholesterol transporter ATP-binding cassette A1 (ABCA1) compared with other monocyte subsets. The cells consequently displayed low cholesterol efflux capacity, avid oxLDL uptake, and potent intracellular production of IL-6, IL-1β, and TNF-α [101]. Interestingly, low levels of cholesterol efflux mediators (such as apolipoprotein A-I (Apo-I) and high-density lipoprotein (HDL) cholesterol) are associated with high CD14++/CD16+ monocyte counts in patients with CKD [101]. Taken as a whole, these observations suggest that the accumulation of CD14+/CD16+ cells may drive endothelium dysfunction, vascular inflammation, and atherosclerosis in CKD. In support of this hypothesis, CD14++/CD16+ monocytes are independently associated with atherosclerotic disease [34] and severe aortic stenosis [36,37] in non-CKD patients, and with cardiovascular events in non-dialysed patients with CKD [39,101]. At present, however, there is no evidence of a direct link between high circulating CD14+/CD16+ monocyte counts and uraemic CVC. In 2016, Yang and colleagues identified CD40 as a potential marker for monocyte activation in patients with CKD [102]. The researchers observed that counts of classical CD40+/CD14++/CD16− and intermediate CD40+/CD14++/CD16+ subsets were abnormally high in patients with CVD and higher still in patients with CVD and CKD. In this study, 37% of the intermediate monocyte subset were CD40+; this proportion was significantly lower in the classical subset (15%) and non-classical subset (28%). The proportion of CD40^+^ monocytes in the intermediate subset was negatively correlated with the estimated glomerular filtration rate (eGFR) and was reported by the authors to express higher levels of the inflammatory markers CD86, HLA-DR, CD11b, CD49d, Ccr2, Ccr5 and Cx3cr1, relative to CD40−/CD14+ monocytes. It remains to be seen whether CD40+ intermediate monocytes are linked to the development of CVC in patients with CKD.

#### 4.1.2. Influence of UTs on Monocyte/Macrophage-Driven CVC

In early stages of CKD, when UTs do not accumulate within the circulation and calcium phosphate metabolism remains unaltered, the role of monocytes/macrophages may be limited to their pro-atherosclerotic actions, as is the case for non-CKD patients. By contrast, in advanced CKD, the elevated levels of circulating UTs may promote atherosclerotic calcification and be responsible for the development of Monckeberg’s medial calcification. Several UTs (notably indoxyl sulphate (IS) and paracresyl sulphate (pCS)) display pro-inflammatory properties, and their serum concentrations are correlated with inflammatory markers in patients with CKD [103]. Some of these inflammatory markers (such as TNF-α, IL-6 and IL-1β) are themselves classified as UTs [104]. A compelling body of evidence suggests that UTs may predispose to CVC through increased monocyte recruitment and direct modulation of the cells’ inflammatory capacities (Table 1 and Table 2). The section below summarizes our current knowledge about this topic. As a reminder, the mean and highest concentrations found in CKD patients of the UTs discussed below are listed in [105].

Phosphate and IS. In patients with moderate CKD, the elevation of serum phosphate concentrations, although still within the normal range (2.5 to 4.5 mg/dL), is associated with a higher prevalence of cardiovascular calcification (coronary artery, descending thoracic aorta and mitral valve calcification) [106]. It has also been observed that in non-haemodialysis CKD patients, the risk of mortality is increased for serum phosphate levels >3.5 mg/dL [107]. In haemodialysis patients, the relative risk of death is increased for serum phosphorus concentrations >5.0 mg/dL [108,109,110]. In clinic, serum IS levels correlate positively with both vascular stiffness and the presence of cardiovascular calcification in patients with CKD (serum IS concentration: 0.17 ± 0.06 mg/100 mL for the 1st tertile, 0.42 ± 0.13 mg/100 mL for the 2nd tertile and 2.11 ± 0.93 mg/100 mL for the 3rd tertile) [111]. In children with CKD, IS is significantly associated with a higher carotid intima-media thickness standard deviation score at baseline and with the progression of central pulse wave velocity deviation score within 12 months (serum IS concentration: 4.2 ± 11.7 µmol/L in CKD stage 3a, 16.5 ± 62.5 µmol/L in CKD stage 3b, 30.7 ± 99.5 µmol/L in CKD stage 4 and 43.9 ± 101 µmol/L in CKD stage 5, *p* < 0.001) [112]. Interestingly, in patients with type 2 diabetes mellitus, serum IS levels are higher in subjects with coronary artery disease (1.0 (0.2–2.1) mg/L) than in patients without coronary artery disease (0.9 (0.5–1.5) mg/L) (*p* = 0.044) [113] and are associated with renal function deterioration, inflammation, and coronary atherosclerosis. In a retrospective analysis conducted in predialysis CKD patients, the use of AST-120 (an oral adsorbent used in the clinic to reduce plasma IS levels) for more than 6 months was shown to be associated with a lower aortic calcification index [114]. Clinical use of AST-120 (6.0 g/day for 24 months) also decreases carotid intima-media thickness and arterial stiffness in undialysed CKD patients [115].

Administration of a high-phosphate diet to CKD mice was associated with greater endothelial expression of adhesion molecules like VCAM-1 and ICAM-1 [116]. In vitro, IS (1.0 mmol/L for 60 min) increased the adhesion of THP-1 monocyte to IL-1β-activated human endothelial cells under physiological flow conditions [117]. In line with this observation, administration of IS to rats (236 mg/mL for 120 min) [118] or mice with normal renal function (0.79 mmol/L released at a rate of 0.5 µL/h for 2 weeks) [119] or to mice with impaired renal function (200 mg/kg/day for 10 days) [120] induced leukocytes to adhere to the vessel wall. The adhesion of leucocytes to inflamed endothelium involves the β2-integrin family of receptors, such as LFA-1 (CD11a/CD18), Mac-1 (CD11b/CD18), p150, 95/CR4 (CD11c/CD18), and CD11d/CD18 [121,122]. The expression of Mac-1 (known to be a receptor for ICAM-1) and ROS production are abnormally high in PBMCs from subtotally nephrectomised CKD mice [117]. In this model, the administration of AST-120 (5% in standard diet for 4 weeks), significantly reduced both Mac-1 expression and the release of ROS—raising the possibility that IS may promote the recruitment of inflammatory leukocytes to the vessel wall. Confirming these data, in adenine-induced uraemic rats, AST-120 (5% in standard diet for 4 weeks) suppressed IS elevation as well as the increase in monocyte adhesion induced by adenine [123]. In vitro, IS (250 µmol/L) promotes the senescence of human large-vessel endothelial cells through the activation of p53 and the production of ROS [124]. Indoxyl sulphate (250 µg/mL) also disrupts contact between bovine pulmonary artery endothelial cells via the phosphorylation of myosin light chain kinase and myosin light chains, and ERK1/ERK2 activation [125]—both phenomena that might favour monocyte extravasation. By promoting the expression of adhesion molecules by both endothelial cells and monocytes, Pi and IS may favour monocyte extravasation and subsequent inflammation-induced CVC.

In rats with reduced renal mass, uraemia promoted M1 polarization of macrophages and impaired M2 polarization by inhibiting adenosine monophosphate (AMP)-activated protein kinase (AMPK) [126]. Hence, the accumulation of UTs within cardiovascular tissues might influence the polarization of infiltrated monocytes/macrophages. In PBMC-derived human primary macrophages, IS (0.5 to 2.0 mmol/L) induced IL-1β, TNF-α and MCP-1 expression in a concentration-dependent manner but had no substantial effects on mRNA levels of anti-inflammatory molecules [127]. Silencing of the *Slco2b1* gene (coding for organic anion transporting polypeptide 2B1 (OATP2B1)) suppressed IS-induced IL-1β and MCP-1 expression in both mouse and human macrophages, suggesting that IS-induced pro-inflammatory macrophage activation is mediated by IS uptake through OATP2B1. In vitro, IS rapidly induces the Notch pathway ligand Dll4 in macrophages by inhibiting the ubiquitin–proteasome pathway, and triggers Notch signalling. In CKD mice, Dll4 blockade abrogated IS (100 mg/kg/day for 7 days)-induced macrophage inflammation and thereby reduced atherosclerosis and calcification [127]. In line with this observation, IS (1 mmol/L) directly induced inflammation as well as ROS production in THP-1 monocytes via the NADPH oxidase and MAPK pathways [117]. Interestingly, the uptake of IS (1 mmol/L) via the aryl hydrocarbon receptor induced pro-IL-1β expression in macrophages differentiated from THP1 cells; this phenomenon was not linked to NLRP3 inflammasome activation but was associated with the subsequent activation of NF-kB and MAPK pathways [128]. These IS-induced inflammatory reactions were associated with low cholesterol efflux in macrophages cultured in vitro [129], suggesting that IS increases lipid accumulation within the cardiovascular wall. It remains to be seen whether IS retention is linked to the low cholesterol efflux capacity of CD14++/CD16+ monocytes isolated from patients with CKD [101]. In THP1-derived macrophages, IS (10 or 20 µM) induced an elevation of M1 (IL-6, CCL2, COX2) and M2 (IL-10, PPARγ, TGF-β, and TIMP-1) markers, giving rise to profibrotic inflammatory macrophages [130]. The calcific potential of these macrophages has not been studied. Overall, Pi/IS-induced monocyte recruitment and IS-induced inflammation, lipid accumulation and fibrosis may be major causes of late-stage calcified atherosclerotic plaques and calcific aortic valve disease in patients with CKD.

**Figure 2:**
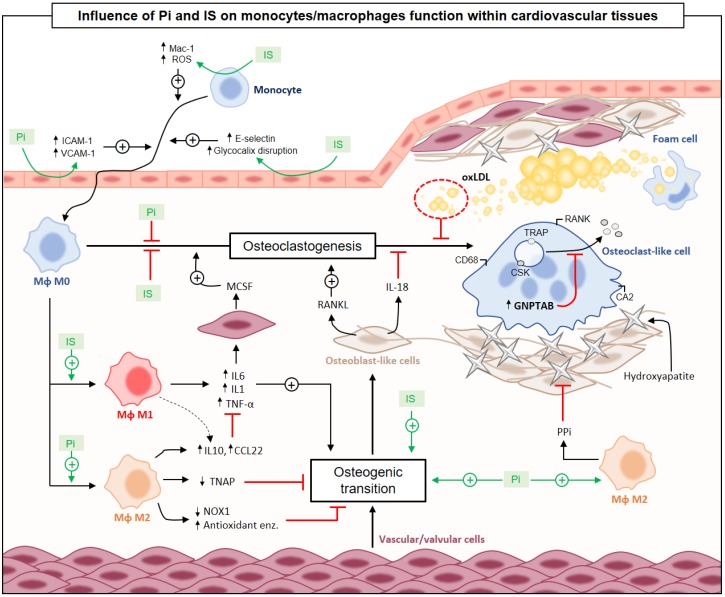
Impact of Pi and IS on monocyte/macrophage function within cardiovascular tissues. Phosphate and IS induce both monocytes and endothelial cells to express adhesion molecules favouring monocyte adhesion, rolling and extravasation into cardiovascular tissues. The exposure of infiltrated monocytes/macrophages to the IS accumulated within cardiovascular structures promotes the cells’ polarization toward a pro-inflammatory phenotype characterized by increased expression of TNF-α, IL-1β, IL-6, and MCP-1. Indoxyl sulphate also prompts monocytes/macrophages to express M2 markers such as IL-10 and TGF-β, giving rise to profibrotic inflammatory macrophages. In contrast, unpolarized macrophages adopt a phenotype similar to that of M2 macrophages in response to Pi. The latter macrophages have anticalcific properties mediated by the greater availability of extracellular ATP and PPi, greater antioxidant synthesis, and lower levels of TNAP, suggesting the existence of a compensatory mechanism that protects tissues from pathologic calcifications linked to high serum phosphate levels. Lastly, Pi and IS inhibit monocyte differentiation into osteoclasts in vitro and their capacity to resorb the bone. A similar effect has been observed with oxLDL. Although the uraemic milieu stimulates CVC by promoting osteoblast-like cells formation, it may also inhibit the resorption of cardiovascular Ca/P nanocrystals by reducing osteoclastic differentiation. CA2: carbonic anhydrase, CSK: cathepsin K, GNPTAB: N-acetylglucosamine-1-phosphate transferase, alpha and beta subunit, ICAM-1: intercellular adhesion molecule, IL-1β: interleukin-1β, IL-6: interleukin-6, IL-10: interleukin-10, IL-18: interleukin-18, IS: indoxyl sulphate, Mac-1: macrophage-1 antigen, MCSF: macrophage colony-stimulating factor, Mφ M0: unpolarized M0 macrophages, Mφ M1: classically-activated macrophages, Mφ M2: alternatively-activated macrophages, NOX: NADPH oxidase, oxLDL: oxidized low-density lipoprotein, Pi: inorganic phosphate, PPi: pyrophosphate, RANKL: receptor activator of nuclear factor kappa-B ligand, ROS: reactive oxygen species, TGF-β: transforming growth factor β, TNAP: tissue-nonspecific alkaline phosphatase, TNF-α: tumour necrosis factor α, TRAP: tartrate-resistant acid phosphatase, VCAM-1: vascular cell adhesion molecule 1.

It is noteworthy that UTs like Pi (1.5 to 4.5 mmol/L) and IS (at mean (211 µmol/L) and maximum (940 µmol/L) uraemic concentrations) block the differentiation of monocytes into osteoclasts and decrease their capacity to resorb the bone in vitro [131,132]. Therefore, while promoting CVC via the formation of osteoblast-like cells, the uraemic milieu may also inhibit the resorption of vascular Ca/P nanocrystals by reducing macrophages differentiation toward osteoclast-like cells. On the same lines, it was recently demonstrated that Pi (2.5 mmol/L) induces murine BMDMs to adopt a phenotype closely similar to that of M2 macrophages [133]. These macrophages displayed an anti-calcific action that was mediated by the greater availability of extracellular ATP and PPi, elevated antioxidant synthesis, and low levels of TNAP; these observations suggest the existence of a compensatory mechanism that protects tissues from pathologic calcifications linked to high serum phosphate levels. The effects mediated by Pi and IS are represented schematically in Figure 2.

Paracresyl sulphate is a metabolite of p-cresol produced by intestinal bacteria. In patients on haemodialysis (HD), serum pCS concentrations are associated with the occurrence and the progression of carotid atherosclerotic plaques (serum pCS levels: 11.60 (3.11–24.70) µg/mL in no carotid atherosclerotic plaque group versus 23.60 (8.62–44.05) µg/mL in carotid atherosclerotic plaque group) [134]. In stable angina patients with early stage renal failure, the median serum total pCS level is significantly higher in subjects with coronary artery disease than in subjects without coronary artery disease (1.7 mg/L (interquartile range 1.0–6.3) vs. 1.0 mg/L (interquartile range 1.0–2.4), *p* *=* 0.008) [135]. In the same manner, in patients with type 2 diabetes mellitus, the serum total pCS level is higher in subjects with coronary artery disease than in subjects without coronary artery disease (2.7 (1.0–7.6) mg/L vs. 1.7 (1.0–5.3) mg/L (*p* = 0.025)) [113]. This increased pCS level is associated with renal function deterioration, inflammation, and coronary atherosclerosis. In elderly haemodialysis patients, a high serum level of free PCS is independently associated with increased risk of all-cause and cardiovascular mortality (free serum pCS levels: 2.7 (range: 0.01–25.6) mg/L in non survivors vs. 1.4 (range: 0.01–10.9) mg/L in control subjects (*p* < 0.001)). According to the study, the total PCS level in this population is 34.7 (range: 1–84.7) mg/L in non-survivors vs. 17.5 (range: 1–48.6) mg/L in the control subjects (*p* = 0.004) [136].

In endothelial cells and macrophages cultured in vitro, pCS (0, 20, 40, 80 µg/mL) promoted the expression of inflammatory factors (TNF-α and MCP-1) and adhesion molecules (ICAM, VCAM, and E-selectin) via NOX activation and ROS production in a concentration-dependent manner; this effect was associated with greater leukocyte-endothelium interactions both in vitro and in vivo [134]. In 5/6-nephrectomized apoE−/− mice, gavage with pCS promoted atherogenesis, and the process was attenuated by NADPH oxidase inhibitors. In these animals, gavage with pCS was associated with the greater expression of adhesion molecules, inflammatory mediators, and NADPH oxidase subunits within the aortic tissue. Therefore, the high levels of pCS observed in patients with CKD might be responsible for the formation of both intimal and medial calcification. These data contradict a previous report in which pCS (63, 250 and 1000 µmol/L) was found to suppress the production of IFN-γ by Th1 cells in vitro [137]. The subsequent observation that pCS (250 and 1000 µmol/L) markedly reduced the expression of IL-12 p70 (the main biological function of which is the induction of IFN-γ in natural killer and T cells) by RAW264.7 cells and primary peritoneal macrophages confirmed this hypothesis [138]. With these cells, exposure to pCS (1000 µmol/L) also enhanced the expression of the anti-inflammatory cytokine IL-10 and suppressed LPS-induced CD40 expression by macrophages. These conflicting results suggest that pCS can negatively regulate Th1-type cellular immune responses by modifying the profile of cytokine secretion in both macrophages and Th1 cells. Further studies will be needed to understand the reasons for these discrepancies. The effects mediated by Pi, IS and pCS are summarized in Table 1.

Guanidino compounds (GCs) form a large group of low-molecular-weight, water-soluble structural metabolites of L-arginine considered to be UTs because of their accumulation in the tissues and biological fluids of patients with CKD [139]. In vitro, exposure to guanidino butyric acid (12.1 µmol/L), guanidino propionic acid (0.5 µmol/L), methylguanidine (24.9 µmol/L), symmetrical dimethylarginine (SDMA) (6.1 µmol/L), asymmetrical dimethylarginine (ADMA) (36.1 µmol/L) or guanidine (13.6 µmol/L) promotes oxidative burst activity in monocytes [140]. The action of SDMA (6.1 µmol/L) on ROS production by monocytes is mediated by Ca^2+^ entry through store-operated channels [141]. The ROS serve as a second messenger, mediating the M1 macrophages’ inflammatory response. In particular, ROS production promotes iNOS and TNF-α after the activation of MAPK and NF-κB, and favours IL1-β production after activation of the inflammasome [142]. These observations suggest that GC-induced ROS may be pro-inflammatory. Indeed, the in vitro exposure of human monocytes to maximal uraemic concentrations of methylguanidine (1.82 µg/mL) and guanidino acetic acid (0.69 µg/mL) was associated with elevated TNF-α production [143]. Schepers and colleagues also reported elevated IL-6 and TNF-α expression in THP1 monocytes cultured in vitro in the presence of SDMA (6.1 µmol/L); this effect was associated with a rise in NF-κB activity [144]. This pro-inflammatory character was further confirmed in patients with CKD, where high levels of SDMA (>1.12 µmol/L) were associated with serum CRP levels [144]. Interestingly, in vitro exposure to ADMA (from 0.6 to 3.6 µmol/L) did not change the inflammatory status of THP1-derived macrophages [144] and guanidino succinic acid (47 µg/mL) inhibited TNF-α production by leucocytes [143], suggesting that GCs have contrasting effects on inflammation. Since oxidative stress and inflammation are key drivers of atherogenesis and the osteogenic transition and subsequent calcification of VSMCs, one cannot rule out the existence of indirect procalcific effects of GCs on the vessel wall via an accentuated oxidative and inflammatory burst in monocyte-macrophages; this question warrants further investigation both in vitro and in animal studies. Indeed, high serum levels of ADMA (≥0.779 µmol/L) predicted the presence of medial calcification in patients with CKD [145]. In addition, serum ADMA activity positively correlated with aortic valve stenosis severity in patients without CKD (serum ADMA activity: 1.94 ± 0.45 µmol/L in patients with severe aortic stenosis vs. 0.87 ± 0.37 µmol/L in patients with mild aortic stenosis (*p* < 0.001)) [146]. In another study of patients with CKD, plasma ADMA levels were negatively correlated with the GFR and positively correlated with coronary artery calcification [147]. In this study, patients with a coronary artery calcification score > 600 showed a mean plasma ADMA value of 0.550 ± 0.078 μmol/L. Plasma levels of SDMA and ADMA were also higher in patients with coronary artery disease (0.62 ± 0.14 vs. 0.74 ± 0.27 μmol/L; *p* = 0.004 and 0.62 ± 0.12 vs. 0.66 ± 0.12 μmol/L; *p* = 0.049, respectively) [148]. Since ADMA (36.1 μmol/L), guanidine (13.6 μmol/L), guanidino acetic acid (5.9 μmol/L), and guanidino butyric acid (12.1 μmol/L) significantly increased the RANKL-induced differentiation of RAW264.7 cells into osteoclast-like cells in vitro [140], one cannot rule out the possibility that GCs modulate the presence and activity of osteoclast-like cells within the vasculature.

Homocysteine (Hcy) and its metabolite S-adenosylhomocysteine (SAH) is a UT that accumulates in the plasma of patients with CKD as a result of impaired extrarenal metabolism [149]. Hyperhomocysteinemia (HHcy) is known to be an independent risk factor for CVD in general and cardiovascular events in CKD in particular [150], which can be used as a biomarker predictive of the cardiovascular prognosis in patients with CKD [151]. In mice, HHcy (plasma Hcy: 213 ± 67.8 µmol/L) accelerated atherosclerosis by promoting the differentiation of bone-marrow- and tissue-derived Ly6C (high) inflammatory monocytes (the murine counterpart of the human inflammatory intermediate monocyte subset) in the aorta and in peripheral tissues [152,153]. This effect was linked to elevated plasma levels of TNF-α and MCP-1, greater monocyte accumulation at the vessel wall, and accentuated macrophage maturation toward the pro-inflammatory M1 phenotype. In this murine model, plasma Hcy levels were positively correlated with plasma levels of pro-inflammatory cytokines. Homocysteine (200 µmol/L) also promoted the inflammatory differentiation of primary mouse splenocytes cultured in vitro [152]. This effect was associated with abnormally low DNA methyltransferase-1 activity and could be reversed by adenoviral DNA methyltransferase-1, suggesting that HHcy-induced DNA hypomethylation is responsible for the differentiation of inflammatory monocytes [152]. Interestingly, exposure to Hcy (10 to 1000 µmol/L) increased the secretion of two potent chemotactic factors (MCP-1 and IL-8) by cultured PBMCs, and this effect was mediated by ROS through NAD(P)H oxidase [154]. In vivo, Hcy-lowering therapy reversed the HHcy-induced differentiation of pro-inflammatory monocytes and the subsequent atherosclerosis [152]. Interestingly, it was recently suggested that Hcy mediates the differentiation of CKD-induced CD40^+^ intermediate monocytes [102]. As mentioned above, this CD40^+^ subtype expresses higher levels of inflammatory markers [102]. In patients with CKD, the plasma Hcy concentration was positively correlated with both CD40^+^ and CD40^+^ intermediate monocyte subset counts, and negatively correlated with the eGFR. In vitro, exposure to either CKD serum (plasma Hcy level: 20.3 µmol/L) or Hcy (100 µmol/L) induced the differentiation of PBMCs into CD40^+^ and CD40^+^ intermediate subtypes [102]. The plasma concentration of soluble CD40L (sCD40L) is elevated in CKD and CVD patients. In vitro, CD40L induced the differentiation of PBMCs into intermediate and CD40^+^ intermediate monocytes. Neutralizing antibodies against CD40L, TNFα or IL-6 prevented the Hcy- or CKD-serum-induced differentiation of PBMCs into CD40^+^ monocytes [102]. S-adenosylhomocysteine is a potent inhibitor of most known methyltransferases [155]. Interestingly, the NFκB consensus element in the CD40 promoter was found to be hypomethylated in white blood cells from patients with CKD and low S-adenosyl methionine (SAM)/SAH ratios [102]. In vitro, exposure to Hcy (100 µmol/L) inhibited DNA methyltransferase-1 activity and promoted the differentiation of PBMCs into CD40^+^ intermediate macrophages—effects that could be reversed by treatment with a remethylating agent such as folic acid. These observations suggest that the elevated levels of the Hcy metabolite SAH (as evidenced by a low SAM/SAH ratio in patients with CKD) are responsible for hypomethylation-induced CD40 expression in monocytes from patients with CKD; as mentioned above, this phenomenon increases the monocytes’ inflammatory potential. Along with Hcy’s well-documented role in the induction of endothelial dysfunction [156], these data suggest that Hcy is a key driver of atherosclerotic, medial and aortic valve calcification. The impact of HHcy-induced pro-inflammatory monocytes/macrophages on the development of CVC has not yet been investigated.

Uric acid (UA) is produced during the metabolism of nucleotides and adenosine triphosphate and constitutes the end product of purine metabolism in humans [157]. In patients under haemodialysis, levels of intimal and medial calcification are independently associated with the serum UA concentration, which in turn is strongly correlated with the serum Pi concentration and the calcium phosphate product (serum uric acid: 6.3 ± 1.0 mg/dL in the non-calcification group versus 7.3 ± 1.2 mg/dL in the arterial medial calcification group (*p* < 0.05) and 6.9 ± 1.2 mg/dL in the arterial intimal calcification group) [45]. In non-CKD patients with asymptomatic hyperuricemia, high serum UA is associated with carotid-intima media thickness (cIMT) [158,159] and increased coronary artery calcification [160,161,162,163]. The presence of silent monosodium urate crystal deposits worsens these calcifications [160]. Although the association between UA and CVC is now well established, the mechanism by which hyperuricemia promotes CVC has yet to be determined. In this context, it is noteworthy that UA directly impacts monocyte/macrophage function. Indeed, the in vitro exposure of human monocytes to a high UA concentration (25 and 50 mg/dL) promoted LPS-induced IL-1β production and inhibited Il-1 receptor antagonist synthesis [164]. Uric acid (12 mg/dL) also promoted the migration and endothelial adhesion of THP-1 monocytes in vitro via greater endothelial expression of MCP-1, IL-8, VCAM-1, and ICAM-1 [165]. On the same lines, in vitro experiments showed that monosodium urate (10 and 20 mg/dL [166] or 500 µg/mL [167]) induced macrophage M1 polarization and NLRP3 inflammasome activation; this effect was associated with the elevated production of IL-1β, and with TNF-α and NF-κB activation [166,167]. The recent observation that allopurinol (an inhibitor of xanthine oxidase, the enzyme responsible for uric acid production) reduced arterial stiffness in mice by producing a relative decrease in vascular oxidative stress, macrophage accumulation and M1 polarization [168] confirmed UA’s key role in vascular inflammation. However, the existence of a causal link between hyperuricemia-induced CVC and UA-induced M1 polarization of monocytes/macrophages has not yet been investigated. In this context, it should be borne in mind that in stage 3 CKD patients with hyperuricemia, an allopurinol-induced lowering of UA levels did not modulate carotid intima-media thickness [169], suggesting that factors other than UA may play a more important role in the regulation of CVC development. Well-designed, prospective, randomized, controlled trials including a larger cohort of patients might help to firmly conclude the effect of UA on CVC in this setting. The various effects of guanidine compounds, Hcy and UA on monocyte/macrophage functions are summarized in Table 2.

Urea is a long-known UT that has long been used as a biomarker of the overall severity of CKD and the adequacy of HD. The recent preclinical and clinical evidence indicates that urea has a number of direct and indirect toxic effects, particularly via the harmful effects of urea-derived carbamylated molecules [170]. Indeed, urea reportedly enhances the carbamylation of lipoproteins—a phenomenon known to promote atherosclerosis [171]. In animals, administration of carbamylated low-density lipoprotein (cLDL) led to the accumulation of cLDL in the vascular endothelium and sub-endothelium. In vitro, exposure to cLDL particles induced the apoptosis of endothelial cells derived from human coronary arteries [172], decreased angiogenesis and the proliferation of endothelial progenitor cells, and accelerated the latter cells’ senescence [173]. Furthermore, exposure in vitro to cLDL particles (i) favours the adhesion of monocytes to endothelial cells by enhancing VCAM-1 and ICAM-1 expression [174], (ii) induces VSMCs proliferation [175], and (iii) favours the accumulation of cholesterol and the formation of foam cells [176]. Carbamylation of HDL abrogates the latter’s cardiovascular protective properties; in vitro, carbamylated HDL fails to promote cholesterol efflux from macrophages [177], impairs angiogenesis, and inhibits endothelial cell migration and proliferation [178]. In this context, the possibility that urea can promote atherosclerotic vascular calcification through macrophage dysfunction and infiltration cannot be ruled out.

### 4.2. Impact of CKD Treatments on Macrophage Functions

Vitamin D supplementation. The majority of patients with ESRD suffer from vitamin D deficiency, and so take vitamin D to prevent secondary hyperparathyroidism. Vitamin D appears to have a complex role in regulating CVC. On one hand, high circulating levels of vitamin D promote ectopic calcification in humans [179]. In addition, elevated vitamin D levels induce CVC in both uraemic and non-uraemic animals [180,181]. These effects are largely due to vitamin D’s stimulatory effect on the intestinal absorption of calcium and Pi; the increased serum mineral levels then predispose to ectopic Ca/P deposition. On the other hand, serum calcitriol levels are inversely correlated with the coronary artery calcification score in the general population [182,183,184]. In line with these data, treatment with vitamin D receptor activators (VDRAs) has been associated with greater cardiovascular survival in several large, cross-sectional studies of patients with CKD [185,186], suggesting the existence of an inhibitory role of vitamin D in CVC development. In accordance with this hypothesis, a recent study reported that murine P388D1 macrophages inhibited VSMC calcification in response to either calcitriol or paricalcitol treatment in a co-culture system [187]. This inhibitory effect was blocked when expression of the vitamin D receptor (VDR) was knocked down prior to co-culture. In the latter study, calcitriol and paricalcitol inhibited macrophage BMP2 and TNF-α expression to the same extent. Activation of the VDR also promoted a 2-fold increase in levels of the calcification inhibitor osteopontin (OPN) in macrophages—a phenomenon that was blocked by the administration of siRNA targeting the VDR. Osteopontin deficiency in macrophages reduced the ability of VDRAs to inhibit SMC mineralization in co-culture—indicating that OPN upregulation and release from co-cultured macrophages is essential for the VDRA-mediated inhibition of VSMCs mineralization. The concomitant decrease in TNF-α expression and increase in OPN expression described in the latter study suggest that VDRAs favour macrophage polarization toward the anti-calcific M2 phenotype [187]. The recent discovery that HD patients with vitamin D levels below 26 ng/ml displayed higher intermediate CD14++/CD16+ monocyte counts than patients with higher vitamin D concentrations (≥26  ng/ml) [188] reinforced the hypothesis whereby dialysed patients with low vitamin D are exposed to a greater risk of inflammatory CVC. Further studies will be needed to confirm or reject this hypothesis.

Haemodialysis. In early studies, the exposure of blood to bio-incompatible dialysis membranes (such as those made of cuprophan or cellulose acetate) or to dialysate endotoxins reportedly caused the activation and apoptosis of circulating monocytes [189,190]. Hence, in an effort to reduce the patient’s inflammatory state, ultrapure dialysates and other highly biocompatible dialysis materials have been introduced. Indeed, the currently used polysulphone-based dialysis membranes are not associated with abnormal circulating counts of classical CD14++/CD16− monocytes, pro-inflammatory intermediate CD14++/CD16+ monocytes, and non-classical CD14+/CD16++ monocytes in HD patients [191]. However, the elevated TLR2 expression and the concomitantly low CD163 expression observed in intermediate monocytes after HD [191] suggests that exposure to dialysis membranes still increases the inflammatory potential of monocytes in this population. Interestingly, the number of CD14+/CD16+ pro-inflammatory monocyte count, the plasma cytokine level, and cytokine mRNA expression levels were significantly lower in patients on haemodiafiltration than in those on HD [192]. This effect may be linked to the use of ultrapure dialysate. At present, ineffective removal of interleukins by conventional high flux (HF) dialyzers seems to be one of the major causes of CVC in subjects with CKD. Indeed, based on in vitro experiments, Zickler and colleagues suggested that high serum levels of TNF-α and IL-6 in subjects on HD are responsible for the CKD-induced osteogenic transition and calcification of VSMCs [193,194]. Conventional dialysis membranes cannot effectively remove inflammatory mediators such as IL-6 and TNF-α [195,196]. Hence, dialysis membranes with a cut-off above 45 kDa have been developed to remove molecules in this size range [197]. In studies of patients on long-term HD, it has been shown that these high cut-off (HCO) dialyzers are associated with lower levels of systemic inflammation than HF dialysis [198,199]. In particular, HCO dialysis cleared β2-microglobulin, sTNF-RI, factor D, and high molecular AGEs better than HF membranes did [200]. The recent report in which exposure to HCO serum markedly lowered the levels of 72 pro-inflammatory transcripts in THP1 cells (including TNF-α and IL-6) compared with exposure to HF serum demonstrated that HCO dialyzers may also directly decrease the inflammatory potential of monocytes and the subsequent calcification of vascular/valvular cells [201]. Indeed, VSMC calcification was 43% lower when the cells were incubated in vitro with HCO dialysis serum, relative to HF dialysis serum; this effect was accompanied by a 26% relative decrease in ALP expression [202]. Unfortunately, HCO dialyzers cause a substantial loss of albumin. Therefore, medium cut-off (MCO) membranes with a sharper cut-off have been recently created [203]. Medium cut-off membranes are associated with lower levels of systemic inflammation in patients with CKD [204]. In a miniature in vitro dialysis model, HCO and MCO lowered plasma levels of IL-6 to the same extent [205]. THP1 cells exposure to HCO or MCO serum did not differ with regard to TNF-α or IL-6 mRNA expression levels [206]. Consequently, HCO and MCO sera appear to be similar in their ability to reduce VSMC calcification and ALP expression in vitro [203]. It is not yet known whether HCO and MCO membranes influence CVC, morbidity and mortality in patients with CKD, relative to HF membranes. Likewise, the membranes’ influence on circulating levels of intermediate monocytes has yet to be investigated.

Calcimimetics. The calcimimetic cinacalcet-HCL (an allosteric modulator of the calcium-sensing receptor (CaSR) expressed by the parathyroid glands) is one of the most effective treatments for secondary hyperparathyroidism. By raising the receptor’s ability to sense extracellular Ca, this calcimimetic decreases serum parathyroid hormone and Ca and P concentrations; this enables better control of secondary hyperparathyroidism in particular and CKD-MBD more generally. Interestingly, the CaSR is expressed in VSMCs [207], where its stimulation by calcimimetics reduces both Pi- and Ca-induced mineralization in cellular models in vitro and in animal models in vivo [208,209,210]. Given the calcimimetics’ systemic effects on the Ca × P product and their local effects on VSMCs mineralization, the clinical use of these compounds was predicted to slow the progression of CVC. To test this hypothesis, the ADVANCE study assessed the progression of cardiovascular calcification in dialysed subjects with secondary hyperparathyroidism taking cinacalcet-HCl [211]. Unfortunately, the primary aortic calcification endpoint (based on the Agatston score) failed to achieve statistical significance. Furthermore, in the randomized controlled “Evaluation of Cinacalcet Hydrochloride Therapy to Lower Cardiovascular Events” (EVOLVE) study of dialysed patients with moderate-to-severe secondary hyperparathyroidism, cinacalcet was not associated with a significantly lower risk of death or major cardiovascular events in a standard intention-to-treat analysis [212]. In this context, the existence of off-target effects of CaSR modulators (thus impairing a reduction in CVC) cannot be ruled out. Peripheral blood monocytes express the CaSR [213,214]. In 2000, Olszak and colleagues noted that the exposure to extracellular calcium or to NPS R-467 (a selective allosteric CaSR activator) increased monocyte chemotaxis in vitro in a concentration-dependent manner. In this model, monocytes derived from CaSR-deficient mice failed to show the normal chemotactic response to a calcium gradient [215]. In the latter study, subcutaneous administration of Ca^2+^ or NPS R-467 favoured the formation of an inflammatory infiltrate consisting of monocytes/macrophages [215], and the effect was amplified by co-administration of MCP1. There is evidence to suggest that CaSR activation in monocytes stimulates the NLRP3-inflammasome system, which in turn mediates the maturation of IL-1β by activating caspase-1 [216]. Indeed, LPS-primed BMDMs release IL-1β in response to extracellular Ca^2+^, gadolinium (a CaSR agonist), and R-568 (a positive allosteric modulator of CaSR) [216,217]. Activation of CaSR in these cells enhanced intracellular Ca^2+^ levels, reduced cyclic AMP levels, and promoted the assembly of the NLRP3 inflammasome components. Extracellular calcium concentrations can increase at sites of infection, inflammation or cell activation. In vitro, the release of extracellular Ca^2+^ from necrotic cells was identified as a danger signal by surrounding monocytes, which promoted NLRP3 inflammasome activation via the phosphatidylinositol/Ca^2+^ pathway; this phenomenon was blocked by CaSR antagonists [217]. In vivo, elevated calcium concentrations amplified the inflammatory response in the mouse model of carrageenan-induced footpad swelling; however, the effect was inhibited in mice lacking GPRC6A, a calcium-responsive group 3 G-protein-coupled receptor closely related to the CaSR [217]. Extracellular calcium may therefore function as an ionic chemokine that promotes inflammation via activation of the CaSR in monocytes. One can reasonably hypothesize that the calcium released by necrotic/apoptotic calcifying VSMCs serves as a chemoattractant for monocytes and thus favours subsequent macrophage-driven inflammation. In this context, it is important to note that in a co-culture system, CaSR/NLRP3 inflammasome activation in M1 macrophages upregulated cardiac fibroblasts’ secretion of MMP-2, MMP-9 and collagen—all factors known to be highly procalcific [218]. It is therefore very likely that in macrophages attracted by necrotic VSMCs, CaSR/NLRP3 inflammasome activation may promote elastin degradation by inducing the remaining viable VSMCs to secrete MMP-2, MMP-9 and collagen.

## 5. Conclusions

Monocytes/macrophages are highly plastic and can adapt their phenotype and functions when facing environmental changes. As demonstrated by the present review, this diversity complicates the pathogenesis of CVC—a disorder for which effective treatments are still lacking. Indeed, the macrophages’ secretion of pro-inflammatory and cytotoxic factors favours the progression of atherosclerotic plaques, while their capacity to remove lipoproteins and apoptotic cells helps to resolve inflammation. Even though macrophages can secrete the osteogenic factor BMP2 under certain conditions, they can also differentiate into osteoclast-like cells (capable of resorbing CVC) under other conditions. These observations suggest that the development of CVC is closely linked to the balance between various macrophage functions. The existence of three subsets of circulating monocytes (i.e., the classical (CD14++/CD16−), intermediate (CD14++/CD16+) and non-classical (CD14+/CD16++) subsets) makes the system even more complex. As discussed above, the intermediate monocyte subset, known as the most inflammatory, is a cellular hallmark of the chronic inflammation associated with CVD in CKD [39] and death in ESRD [219]. It remains to be established whether infiltrated macrophages from the intermediate subset have more procalcific activity than macrophages from the classical or non-classical subsets. Likewise, there is a need for well-designed randomized clinical studies capable of determining whether the increase in circulating CD14+CD16+ monocytes observed in patients on HD is correlated with the degree of CVC. The existence of an additional subtype of highly procalcific macrophages, displaying unique microvesicle and microRNA profiles, and containing scaffolds for calcification, has been suggested [77]; the recently discovered intermediate CD40+CD14++CD16+ subset of monocytes may be a promising candidate because the cells are highly inflammatory and are present in high numbers in the circulation of CKD patients with CVD. Although data from in vitro and in vivo studies suggest that the accumulation of UTs may promote monocytes/macrophages procalcific properties, to date strong evidence based on clinical data is still missing to clearly establish the existence of a causal link. PTH is one of the UTs associated with the development of cardiovascular calcification in CKD patients [220]. Currently, no information is available concerning the impact of PTH on monocytes/macrophages procalcific functions. This topic needs to be further investigated. Although this review mainly focused on the impact of UTs on monocytes/macrophages procalcific properties, the impact of hormonal and metabolic dysregulations linked to CKD should not be neglected. A compelling body of evidence indicates that in a CKD setting, the removal of UTs with poorly biocompatible dialyzers increases the pro-inflammatory and subsequently procalcific potential of monocytes/macrophages. This important side effect must be taken into consideration. Research on the macrophages’ role in preventing or resorbing arterial mineral deposits is in its early stages. Given that CVC rarely regresses, the major objective of this approach is to prevent calcification. A better understanding of the molecular mechanisms by which macrophages differentiate into osteoclast-like or into osteoblast-like cells will be needed to advance research in this area. In the future, the key issue will be to determine whether and how macrophages can be targeted to prevent excessive CVC in subject with CKD [96]. In particular, it will be important to establish whether macrophages’ anti-calcific activities can be induced without causing the cells to lose their beneficial roles in inflammation resolution or in promoting osteogenic pathways in vascular/valvular cells. Therefore, a better understanding of the role played by macrophage subtypes in atherosclerotic plaque stability and in medial and cardiac valve calcification will be needed before efficient therapeutics can be developed.

## Figures and Tables

**Table 1:** Impact of inorganic phosphate (Pi), indoxyl sulphate (IS) and paracresyl sulphate (pCS) on monocyte/macrophage infiltration and function. AhR: aryl hydrocarbon receptor, AP1: activator protein 1, ApoE: apolipoprotein E, ARG-1: arginase 1, CCL2: C-C motif 22, Cox2: cyclooxygenase 2, eNTPD1: ectonucleoside triphosphate diphosphohydrolase 1, HAEC: human aortic endothelial cell, HIF-1: hypoxia-inducible factor 1, HUVEC: human umbilical vein endothelial cell, ICAM-1: intercellular adhesion molecule, IFN-γ: interferon γ, IL-1β: interleukin-1β, IL-4: interleukin-4, IL-6: interleukin-6, IL-10: interleukin-10, JNK: c-Jun N-terminal kinase, LDLR: low-density lipoprotein receptor, Mac-1: macrophage-1 antigen, MAPK: mitogen-activated protein kinase, MCP1: monocyte chemoattractant protein 1, MLC: myosin light chain, MLCK: myosin light chain kinase, NFΚB: nuclear factor-kappa B, NOX: NADPH oxidase, Nrf2: nuclear factor erythroid-2-related factor 2, PBMC: peripheral blood mononuclear cell, PGC1β: peroxisome proliferator activator receptor γ coactivator-1β, PPARγ: peroxisome proliferator-activated receptor γ, PPi: pyrophosphate, RANKL: receptor activator of nuclear factor kappa-B ligand, ROS: reactive oxygen species, Th1: T helper cell type 1, Th2: T helper cell type 2, TIMP-1: tissue inhibitor of metalloproteinase 1, TNAP: tissue-nonspecific alkaline phosphatase, TNF-α: tumour necrosis factor α, TRAP: tartrate-resistant acid phosphatase, VCAM-1: vascular cell adhesion molecule 1. ND: not documented.

Uraemic Toxins	Action	Signalling	Experimental Model	Potential Effect on CVC	Ref
Phosphate	VCAM ICAM	ND	CKD mice	Procalcific	[116]
	Osteoclast differentiation TRAP resorption	RANKL-induced NFΚB, AP1 and Sp1/Sp3 via Na/Pi co-transporters	PBMC, RAW 264.7	Procalcific	[131]
	ARG1 and arginine degradation PGC1β, HIF-1 NOX1 Antioxidant enzymes, antioxidant metabolites ATP, PPi eNTPD1, TNAP	ND	Mice BMDMs	Anticalcific	[133]
Indoxyl sulphate	MAC1 ROS	P38 phosphorylation Translocation of NADPH oxidase subunit p47 phox	PBMCs from CKD mice	Procalcific	[117]
	THP1 adhesion to IL1-β-activated HUVECs		THP1 and HUVECs		
	Adhesion, extravasation, glycocalix disruption	ND	Rat circulating leukocytes	Procalcific	[118]
	TNF-α-induced leukocyte adhesion though E-selectin	Intake via AhR AP1 activity	Non-CKD mice	Procalcific	[119]
		JNK, P38 and NFΚB NADPH oxidase	THP1 and HUVECs CKD mice	Procalcific	[120]
	Endothelial cell senescence	ROS and P53	HUVECs	Procalcific	[124]
	Adherens junction between endothelial cells	ROS which activates ERK1/2 pathway and subsequent MLCK and MLC phosphorylation	Bovine pulmonary artery endothelial cells	Procalcific	[125]
	IL-1β, TNF-α and MCP1	Ubiquitin proteasome pathway Notch signalling	PBMCs Ldlr-/- mice with CKD	Procalcific	[127]
	Pro-IL1β	Intake via AhR NFΚB and MAPK activation	THP1-derived macrophages	Procalcific	[128]
	Polarization toward low inflammatory pro-fibrotic macrophages: IL-6, CCL2, Cox2 CD163, IL-10, PPARγ, TIMP1	Intake via Ahr Nrf2 activation	THP1	Procalcific	[130]
	Viability, cholesterol efflux IL-1β, TNF-α and ROS	ND	THP1-derived macrophages	Procalcific	[129]
	Osteoclast differentiation, resorption	JNK, P38, AKT, ERK1/2 DNA binding activity of AP1 and NFΚB	RAW 264.7 and PBMCs	Procalcific	[132]
Paracresyl sulphate	TNF-α, MCP1 and ROS Monocytes/endothelial cells interactions Atherogenesis and infiltration E-selectin, ICAM-1 and VCAM-1	Nox1, Nox4 and P22	HUVECs, HAEC, THP1 and peritoneal macrophages ApoE-/- mice with CKD	Procalcific	[134]
	IFN-γ, IL-4 Th1 cells, Th2 cells	ND	Mouse splenocytes	Anticalcific	[137]
	IL12 p70, IL-10, CD40	ND	RAW 264.7 and peritoneal macrophages	Anticalcific	[138]

**Table 2:** Impact of guanidino compounds, homocysteine, and uric acid on monocyte/macrophage infiltration and function. ADMA: asymmetrical dimethylarginine, ApoE: apolipoprotein E, BMDM: bone marrow-derived macrophage, CBS: cystathionine beta-synthase, G: guanidine, GAA: guanidino acetic acid, GBA: guanidino butyric acid, GPA: guanidino propionic acid, GSA: guanidino succinic acid, HUVEC: human umbilical vein endothelial cell, ICAM-1: intercellular adhesion molecule, IL-1β: interleukin-1β, IL-1RA: interleukin-1 receptor antagonist, IL-6: interleukin-6, IL-8: interleukin-8, TNF-α: tumour necrosis factor α, MCP1: monocyte chemoattractant protein 1, MG: methylguanidine, M1: classically-activated macrophage, M2: alternatively-activated macrophage, mTOR: mammalian target of rapamycin, NFΚB: nuclear factor-kappa B, PARP: poly(ADP-ribose) polymerase, PBMC: peripheral blood mononuclear cell, PKC: protein kinase C, SOC: store-operated channel, RANKL: receptor activator of nuclear factor kappa-B ligand, ROS: reactive oxygen species, SDMA: symmetrical dimethylarginine, VCAM-1: vascular cell adhesion molecule 1. ND: not documented.

Uraemic Toxins		Action	Signalling	Experimental Model	Potential Effect on CVC	Refs
**Guanidino compounds**	SDMA	ROS	Ca^2+^-entry via SOCS	Human PBMCs	Procalcific	[140,141]
		IL-6 and TNF-α	NFΚB pathway	THP1	Procalcific	[144]
	GPA	ROS	ND	Human monocytes	Procalcific	[140]
	MG	TNF-α	ND	Human monocytes	Procalcific	[143]
		ROS	ND	Human monocytes	Procalcific	[140]
	GAA	TNF-α	ND	Human monocytes	Procalcific	[143]
		RANK-L induced osteoclastogenesis	ND	RAW 264.7	Anticalcific	[140]
	ADMA	Endothelial cell senescence	ROS and P53	HUVECs	Procalcific	[124]
		No impact on inflammation	ND	THP1	None	[144]
		RANK-L induced osteoclastogenesis	ND	RAW 264.7	Anticalcific	[140]
		ROS	ND	Human monocytes	Procalcific	[140]
	GSA	TNF-α	ND	Human monocytes	Anticalcific	[143]
	GBA	RANK-L induced osteoclastogenesis	ND	RAW 264.7	Anticalcific	[140]
		ROS	ND	Human monocytes	Procalcific	[140]
	G	RANK-L induced osteoclastogenesis	ND	RAW 264.7	Anticalcific	[140]
		ROS	ND	Human monocytes	Procalcific	[140]
Homocysteine		IL-8 and MCP1	PKC/calmodulin NADPH oxidase, ROS p38, ERK1/2 and NFΚB activation	PBMCs	Procalcific	[154]
		Ly-6C subset accumulation within atherosclerotic lesions MCP1, TNF-α	NADPH oxidase-mediated oxidative stress	Tg-hCBS apoE^-/-^ Cbs^-/-^ mice	Procalcific	[153]
		Atherosclerotic lesions Ly-6C subset TNF-α M1 and M2 polarization	DNA methyltransferase activity	Tg-hCBS apoE^-/-^ Cbs^-/-^ mice Primary mice splenocytes	Procalcific	[152]
		CD40 / CD40 intermediate monocytes	DNA methyltransferase activity DNA hypomethylation of the NFΚB consensus element in the CD40 promoter	PBMCs	Procalcific	[102]
		Endothelial cell apoptosis	ROS Transmembrane mitochondrial potential Cytochrome C release Caspase 3 and 9 activation, PARP cleavage	Microvascular endothelial cells (MVECs)	Procalcific	[156]
Uric acid		IL1RA IL1-β	AKT/PRAS40 pathway mTOR signalling Autophagy through LC3I / LC3II	PBMCs	Procalcific	[164]
		Viability MCP1, Il-8, VCAM-1, ICAM-1 THP1 migration and adhesion to HUVECs	NFΚB activation	THP1, HUVECs Rats with hyperuricemia	Procalcific	[165]
